# Antitumor effect of therapeutic HPV DNA vaccines with chitosan-based nanodelivery systems

**DOI:** 10.1186/s12929-014-0069-z

**Published:** 2014-07-31

**Authors:** Alireza Tahamtan, Amir Ghaemi, Ali Gorji, Hamid R Kalhor, Azadeh Sajadian, Alijan Tabarraei, Abdolvahab Moradi, Fatemeh Atyabi, Mishar Kelishadi

**Affiliations:** 1 Department of Microbiology, Golestan University of Medical Sciences, Gorgan, Iran; 2 Department of Virology, School of Public Health, Tehran University of Medical Sciences, Tehran, Iran; 3 Shefa Neuroscience Research Centre, Tehran, Iran; 4 Institut für Physiologie I, Westfälische Wilhelms-Universität Münster, Robert-Koch-Strasse, Münster, 48149, Germany; 5 Klinik und Poliklinik für Neurochirurgie, Westfälische Wilhelms-Universität Münster, Münster 48149, Germany; 6 Department of neurology, Westfälische Wilhelms-Universität Münster, Münster, Germany; 7 Biochemistry Research laboratory, Department of Chemistry, Sharif University of Technology, Tehran, Iran; 8 Nanotechnology Research Centre, Faculty of Pharmacy, Tehran University of Medical Sciences, Tehran, Iran

**Keywords:** Human papilloma virus, E7, DNA vaccine, Chitosan, Tumor

## Abstract

**Background:**

Cervical cancer is the second-most-common cause of malignancies in women worldwide, and the oncogenic activity of the human papilloma virus types (HPV) E7 protein has a crucial role in anogenital tumors. In this study, we have designed a therapeutic vaccine based on chitosan nanodelivery systems to deliver HPV-16 E7 DNA vaccine, considered as a tumor specific antigen for immunotherapy of HPV-associated cervical cancer. We have developed a Nano-chitosan (NCS) as a carrier system for intramuscular administration using a recombinant DNA vaccine expressing HPV-16 E7 (NCS-DNA E7 vaccine). NCS were characterized in vitro for their gene transfection ability.

**Results:**

The transfection of CS-pEGFP NPs was efficient in CHO cells and the expression of green fluorescent proteins was well observed. In addition, NCS-DNA E7 vaccine induced the strongest E7-specific CD8+ T cell and interferon γ responses in C57BL/6 mice. Mice vaccinated with NCS-DNA E7 vaccine were able to generate potent protective and therapeutic antitumor effects against challenge with E7-expressing tumor cell line, TC-1.

**Conclusions:**

The strong therapeutic effect induced by the Chitosan-based nanodelivery suggest that nanoparticles may be an efficient carrier to improve the immunogenicity of DNA vaccination upon intramuscular administration and the platform could be further exploited as a potential cancer vaccine candidate in humans.

## Background

Cervical cancer is the most common cancer in women worldwide and the leading cause of death from cancer among women in the developing countries of the world [1],[2]. HPV-16 is the predominant etiologic agent of cervical cancer and encodes three transforming oncogenes, E5, E6, and E7. Their products are thus unique tumor antigens and can be used as tumor vaccines [3]. Because E6 and E7 oncoproteins are consistently retained and expressed, the E6 and E7 oncoproteins represent natural targets for anti-tumor immune response, and therefore are considered tumor-associated antigens (TAA) [4].

Several approaches have been used to develop HPV therapeutic vaccines including live vector, peptide/protein, DNA vaccine and whole cell-based approaches. These studies have shown the importance of T-cell responses in protecting against tumors in human and animal models [5]. Of these approaches, DNA vaccine targeting the E7 antigen offers a potentially effective approach to control E7-expressing tumors [4],[6],[7].

DNA vaccines represent a valid approach to the generation of antigen-specific immunotherapy for several reasons [8],[9]. The plasmid vectors are safe, have low immunogenicity, and can be repeatedly administered. Additionally, DNA vaccines can be easily prepared in large scale with high purity and are highly stable compared to viral vectors [10]. DNA vaccines have also demonstrated to generate both humoral and cell mediated immune responses [11],[12].

However, there are several drawbacks preventing the clinical application of DNA vaccines [13]. After intramuscular administration, it is difficult for the vaccines to move through cell membranes, so only a small amount reaches antigen presenting cells (APC) to induce immune responses [14],[15].

Although the reasons for lack of potency of DNA vaccines in human subjects and non-human primates remain poorly understood [10], there is a clear need to improve the transfection efficiency in vivo for allowing much lower dose of plasmid DNA.

Novel delivery systems for administration may be keys to address this need. Among possible delivery systems, chitosan nanoparticles hold promise because of their ability to protect encapsulated nucleic acid-based antigens, and to promote delivery of adsorbed DNA to antigen-presenting cells [13],[16].

Chitosan (CS) is a non-toxic biodegradable and biocompatible polycationic polymer, which can bind and protect the entrapped DNA from degradation by nuclease and increase the efficiency of cellular DNA uptake [16]-[18], additionally CS exhibit potential adjuvant properties, such as promoting endocytotic uptake and elevating immune responses [19].

Zaharoff et al. have demonstrated that chitosan solution improved antigen-specific antibody and antigen-specific splenic CD4+ proliferation responses to a subcutaneous vaccination with a model protein antigen in the absence of additional adjuvants [20]. These studies established a basis for the use of chitosan nanoparticles as a DNA vaccine delivery system.

In this study, we have evaluated the immune response elicited in C57BL/6 mice by Nano-chitosan (NCS) containing a recombinant DNA vaccine expressing HPV-16 E7, as well as the levels of protection against TC-1 tumor cell challenge, following intramuscular administration.

## Methods

### Mice and cells

Female 6-8-week-old C57BL/6 mice were obtained from the Institute Pasteur of Iran (Karaj, Iran). Mice were housed for 1 week before the experiment, given free access to food and water and maintained in a light/dark cycle. All experiments were carried out in accordance with the Animal care and use protocol of Golestan University of Medical Sciences of Iran.

The production and maintenance of TC-1 cells have been described previously [12]. The cells were C57BL/6 mouse lung endothelial cells transformed with HPV 16 E6 and E7 oncogenes and an activated H-ras gene.

The in vitro transient transfection studies were performed in Chinese hamster ovary (CHO) (Pasteur Institute of Iran) cell line. The TC-1 and CHO cell lines were cultured in RPMI 1640 medium (Gibco-BRL, UK) supplemented with 10% FBS (Gibco), 1 mM sodium pyruvate (Sigma), 100 U/ml penicillin, and 100 μg/ml streptomycin, 0.4 mg/ml G418, and 0.2 mg/ml hygromycin at pH 7.2 at 37°C with 5% CO_2_.

### Plasmid construction

The generation of pcDNA3.1-E7 has been described previously. Plasmid constructs were confirmed by DNA sequencing and expression. Amplification and purification of DNA were previously described [6]. Stocks of endotoxin free DNA vaccine plasmids and vector control plasmid (pcDNA3.1) in 0.1 M PBS were prepared for in-vivo immunization studies using the EndoFree® Plasmid Maxi Kit (Qiagen, Hilden, Germany) and dissolved in endotoxin-free Tris-EDTA (Sigma, St. Louis, MO).

### Preparation and characterization of nano-chitosan-based DNA vaccine

Chitosan (CS) with the deacetylation degree of 95% and the molecular weight (MW) of 360 kDa was purchased from the Primex (Karmoy, Norway) and depolymerized by a chemical reaction as previously described [21] to obtain the low molecular weights of CS. The molecular weight of depolymerized CS was determined by gel permeation chromatography (GPC) using pullulane standards [22]. The Nano-chitosan (NCS) were prepared according to the ionotropic gelation method based on the interaction between the negative groups of the pentasodium tripolyphosphate (TPP) cross-linker (Merck, Darmstadt Germany) and the positively charged amino groups of NCS as described previously [23]. The complexes were allowed to stand at room temperature for 30 min and stored at 4°C until use.

The hydrodynamic mean diameter of prepared chitosan nanoparticles was determined by dynamic light scattering using Nano-Zetasizer (Malvern Instruments, Worcestershire, United Kingdom) with a wavelength of 633 nm at 25°C with an angle detection of 90 degrees. The samples were diluted with freshly filtered deionized water and all measurement was done three times. The zeta potential of prepared chitosan nanoparticles was determined by laser Doppler electrophoresis using Zetasizer (Nano-ZS Malvern Instruments). All samples were diluted (1:5) in deionized water and each sample was measured three times.

NCS -DNA vaccine complex were prepared as previously described [24]. Briefly, the complex formation between NCS and pDNA at different N/P ratios were analyzed by 0.8% agarose gel electrophoresis in TBE buffer along with naked pDNA as control. Sample was analyzed using electrophoresis at 40 V for 60 min. The in vitro transfection study was performed using CHO cell line and pEGFP-N1 (Clontech Laboratories) as marker gene. EGFP expression in cells investigated through fluorescence microscopy. To evaluate the expression of NCS-DNA vaccine encoding HPV-16 E7 gene (NCS-DNA E7) in the CHO cells, SDS-PAGE and Western blot analysis using monoclonal mouse anti-HPV E7 antibody were used. Briefly, the extracted total proteins lysed in SDS-PAGE sample loading buffer, and lysates separated by SDS-PAGE. Separated proteins by SDS-PAGE, transferred onto PVDF, and E7 protein was detected using anti-E7 monoclonal antibody (Abcam, UK). The specific band was detected with goat anti-mouse secondary antibody (Sigma, St Louis, MO) and staining with diaminobenzidine (DAB) substrate. The TC-1 cell line and cells transfected with Lipofectamin were used as controls.

### Tumor therapy assay

For in vivo therapeutic experiments, C57BL/6 mice were divided into six groups (n = 10). The mice were challenged by subcutaneous (S.C) injection in the right flank with 2×10^5^ TC-1 cells suspended in 100 μl PBS. After one week, the mice were immunized intramuscularly with 90 μg naked DNA vaccine encoding HPV-16 E7 (DNA-E7) thrice at 7-day intervals (group 1).

Each animal in Groups II and III were respectively administered with 90 μg NCS-DNA E7 and NCS-pcDNA3.1 plasmid with the same protocol.

Blank Nano-chitosan (NCS), PBS and pcDNA3.1 were injected according to the same protocol into the IV, V and VI group of mice as control groups (NCS, PBS and pcDNA_3_ groups).

Subcutaneous tumor volume was estimated according to Carlsson’s formula [12]. Hence, the largest (a) and the smallest (b) superficial diameters of the tumor were measured in a blinded, coded fashion twice a week and then the volume (V) of the tumor was calculated (V = a × b × b/2). Statistical analysis was performed using Student’s *t* test. All values were expressed as means ± S.D.

Five mice per group were sacrificed one week following the third immunization and the spleens were removed aseptically, and then cell proliferation, cytolytic activity and cytokine secretion were assayed. All tests were performed in triplicate for each mouse. Results are representative of three independent experiments.

### Lymphocyte proliferation assay (LPA)

One week after the third immunization, 5 mice per group were sacrificed and their splenocytes were obtained and treated with ammonium chloride-potassium lysis buffer for 1 min to deplete erythrocytes. In 96-well flat-bottom culture plates (Nunc, Denmark) splenocytes (2 × 10^5^ per well) were cultured in triplicate with RPMI-1640 supplemented with 10% fetal calf serum, 1% L-glutamine, 1% HEPES, 0.1% penicillin/streptomycin. The cultured cells were incubated with 4 × 10^5^ TC-1 cells previously treated with mitomycin C (Sigma, St. Louis, Mo) (30 μg/ml for 3 h), T cell mitogen PHA (phytohemagglutinin, positive control), 2 μg/ml of BSA (irrelevant antigen), or medium (negative control) per well at 37°C in 5% CO_2_.

After 3 days, MTT (3-(4,5-dimethyl tetrazolyl-2) 2,5 diphenyl) tetrazolyumbromide (Sigma chemicals) in concentration of 5 μg/ml was added per well and incubated for 5 h at 37°C in 5% CO_2_. DMSO (dimethyl sulfoxide) (100 μl) was added to dissolve produced formazan crystals.

Plates were read at 540 nm, and the results were expressed as stimulation index (SI). The SI was determined as follows: OD values of stimulated cells minus relative cell numbers of unstimulated cells divided by relative OD values of unstimulated cells.

All tests were performed in triplicate for each mouse.

### CD8 cytotoxicity assay

One week after the third immunization, for each sample obtained from individual mouse (five mice per group), single cell suspension of mononuclear cells (used as the effecter cells) were cocultured in phenol red-free RPMI containing 3% FCS with washed target cells EL4 at different effector-to-target cell (E/T) ratio (25:1, 50:1 and 100:1). For preparation of the target cells, EL-4 cells were stimulated with 4 × 10^5^ TC-1 cells previously treated with mitomycin C (30 μg/ml for 3 h) and then incubated for 4 h.

After centrifugation, the supernatants (50 μl/well) were transferred to the 96-well flat-bottom plates, and lyses of target cells were determined by measuring LDH release using Cytotoxicity Detection Kit (LDH) according to the procedures stated by the manufacturer (Takara Company).

Several controls were used. The ‘high control’ was used for measuring total LDH release from the target cells (all EL4 cells were lysed with medium containing 1% Triton X-100). The ‘low control’ was used for measuring natural release of LDH from the target cells (which was obtained by adding EL4 cells only to the assay medium).

The optical density was read at 490 nm after 30 min incubation at room temperature and cytotoxicity was calculated according to the formula:

(1) $$\%\;\mathrm{Cytotoxicity}\;=\;\left(\mathrm{Experimental}\;\mathrm{value}\;-\;\mathrm{Low}\;\mathrm{control}\;/\;\mathrm{High}\;\mathrm{control}\;-\;\mathrm{Low}\;\mathrm{control}\right)\times 100$$

### Cytokine assay

One week after third immunization, splenocytes were obtained from five immunized mouse of each group and used for the IFN-γ and IL-4 production assay. Briefly, 5 × 10^5^ cells/well in the presence of 4 × 10^5^ TC-1 cells previously treated with mitomycin C (30 μg/ml for 3 h) were harvested in 96-well flat-bottom plates with complete RPMI 1640 supplement with 10% FCS, 1% L-glutamine, 1% HEPES, 0.1% 2-mercaptoethanol and 0.1% penicillin/streptomycin. Cells were cultured for 2 days at 37°C in 5% CO_2_. Supernatants were then collected and analyzed for the presence of IFN-γ and IL-4 using ELISA commercial kits according to the manufacturers protocol (eBioscience, Inc. San Diego, CA). All tests were performed in triplicate for each mouse (Five mice per group).

### Statistical analysis

Lymphocyte proliferation, CTL and cytokine assay were analyzed by a one-way ANOVA. Significant differences of tumor growth on given days were assessed by Student’s *t*-test. Differences were considered statistically significant when P value <0.05. All tests were performed in triplicate and all data are expressed as mean ± SD.

## Results

### Characterization of chitosan-based nanodelivery systems for E7 DNA vaccine

The particle size of the NCS ranged between 40 and 150 nm; nanoparticles showed a narrow size distribution (PDI: 0.149), positive zeta potential with a mean diameter of about 70 nm.

The mean zeta potential of prepared NPs before packaging was approximately + 20 mV.

NCS/pDNA complexes were developed by adding the pDNA solution to the prepared nanoparticle’s solution. The complex formation was confirmed by gel electrophoresis [24].

**Figure 1:**
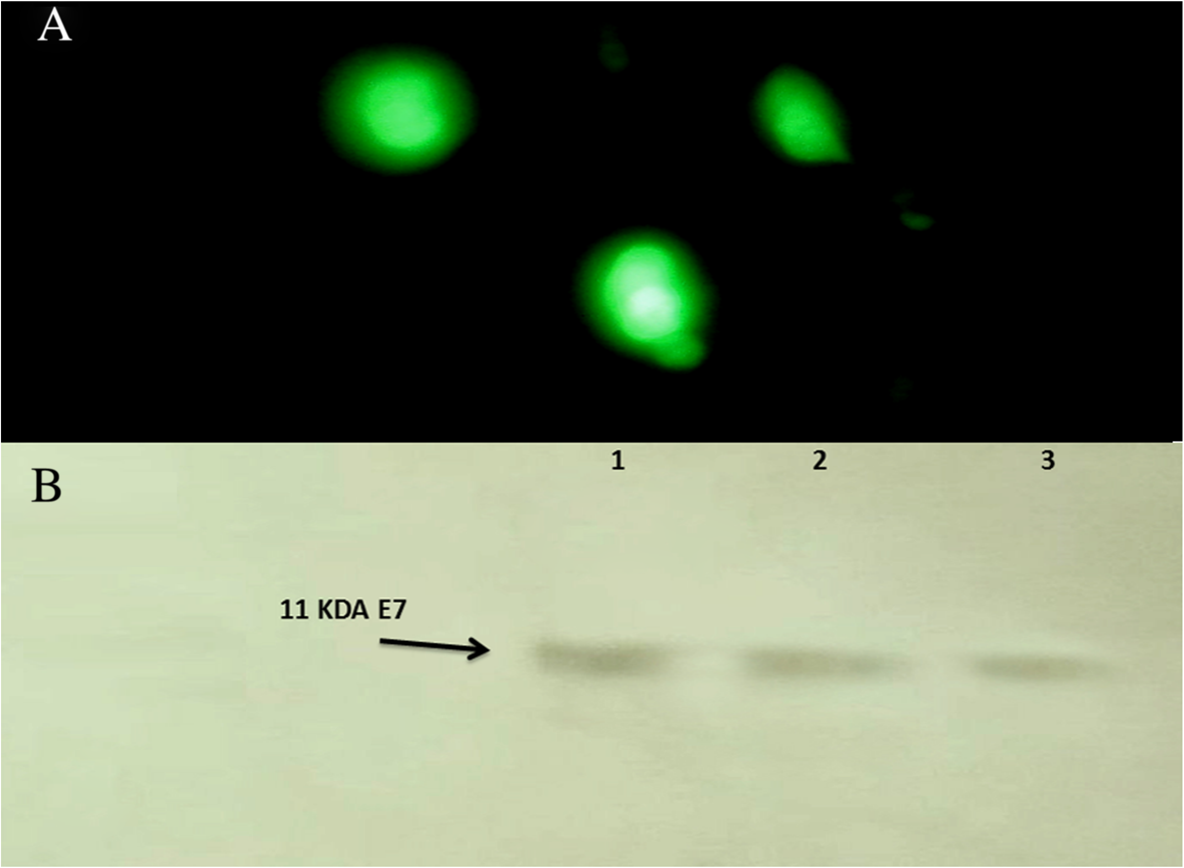
**Transfection analysis of the chitosan nanodelivery systems. A)** CHO cells transfected by NCS – pEGFP; **B)** Western blot analysis of HPV E7 antigen expression by NCS-DNA E7. Cell extracts or supernatants were prepared as described in Methods; proteins were separated by SDS polyacrylamide gel electrophoresis, blotted on PVDF membranes, and incubated with the specific anti-HPV-16 E7 monoclonal antibody. The arrow indicates the E7 protein of approximately 11 KDa of molecule weight. Lane 1: Lysate from TC-1 cells, Lane 2: pcDNA3.1/E7 with Lipofectamine, lane 3: NCS-DNA E7.

The in vitro transfection study was performed using CHO cell line and EGFP-N1 as marker gene through fluorescence microscopy. EGFP expression was observed in CHO cells transfected with NCS – pEGFP in a similar extent to the control cells which were transfected with lipofectamine (Figure 1A).

To evaluate the expression of HPV-16 E7 gene in the CHO cells, Western blot analysis using monoclonal mouse anti-HPV E7 antibody was used. The TC-1 cell line and cells transfected with Lipofectamine were used as positive controls. The analysis showed an expression of HPV-16 E7 11 KDa from NCS-DNA E7 in the Western blot according to the TC-1 cells and cells transfected with Lipofectamine (Figure 1B).

### Lymphocyte proliferation response to E7 antigen

One week after the third immunization, the splenocytes from the immunized mice were harvested and re-stimulated in vitro with E7 antigen for the lymphocyte proliferation assay.

**Figure 2:**
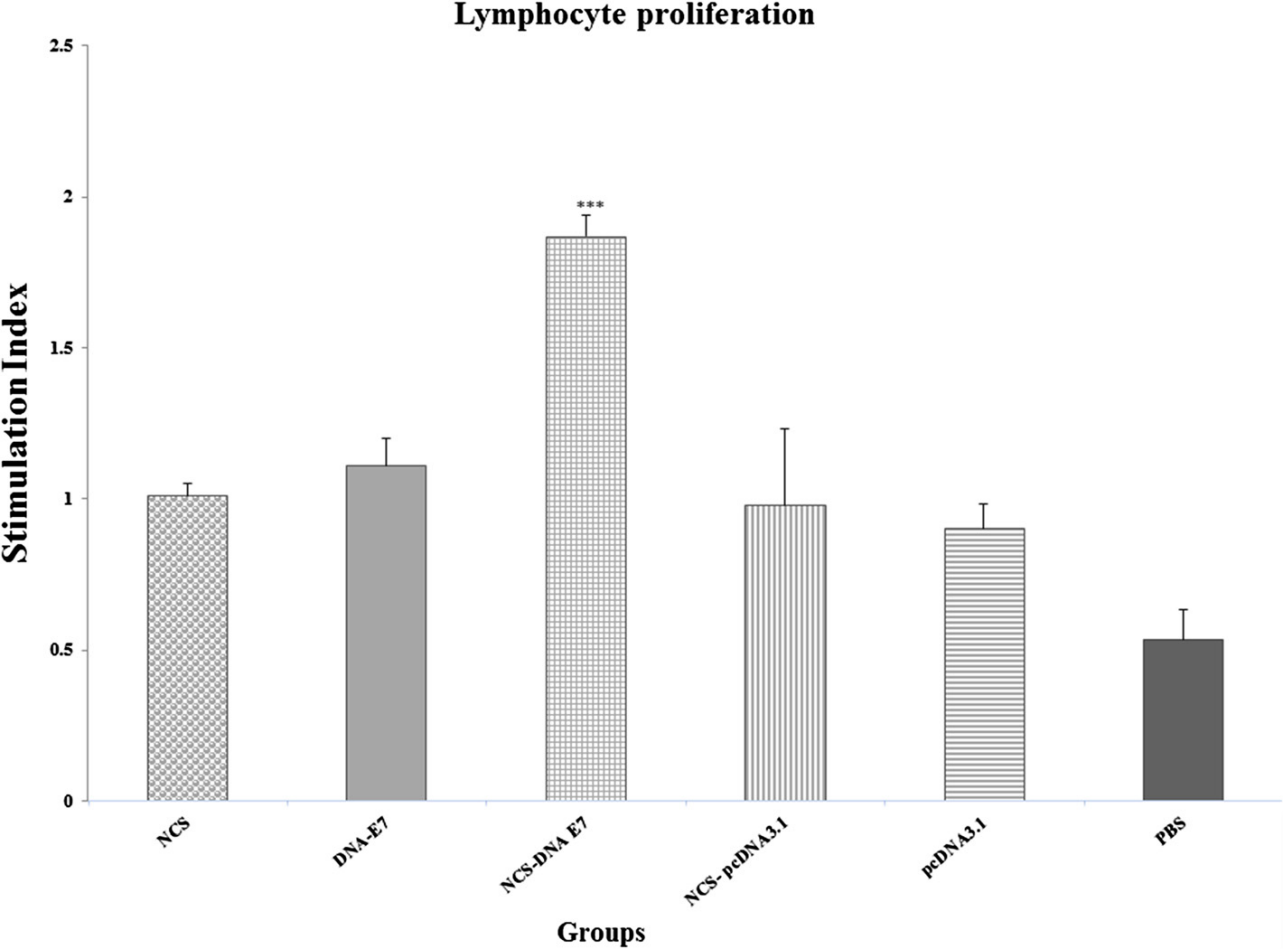
**Splenocyte proliferation levels after in vitro stimulation with HPV-16 E7 antigen.** The mice were injected intramuscularly thrice at 7-day intervals with different Nano-chitosan (NCS) regimes. One week after final immunization, spleens of individual mice (five per group) were removed and lymphocyte proliferation was evaluated with MTT method. Formazan crystals were dissolved in dimethyl sulfoxide and optical densities were read at 540 nm. Values are the mean ± standard error of the mean for the experiments. *** Indicates statistically significant difference between the NCS-DNA E7 group as determined by one-way ANOVA (P < 0.001) with other groups.

As shown in Figure 2, the lymphocyte proliferation was significantly higher in mice treated with the NCS-DNA E7 group than in those treated with DNA-E7 and control groups (DNA-E7, NCS, NCS-pcDNA3.1, PBS and pcDNA3.1) (P <0.001). As it appeared, there is no (statistically) significant difference among control groups.

### Induction of antigen specific CD8+ CTL activity

**Figure 3:**
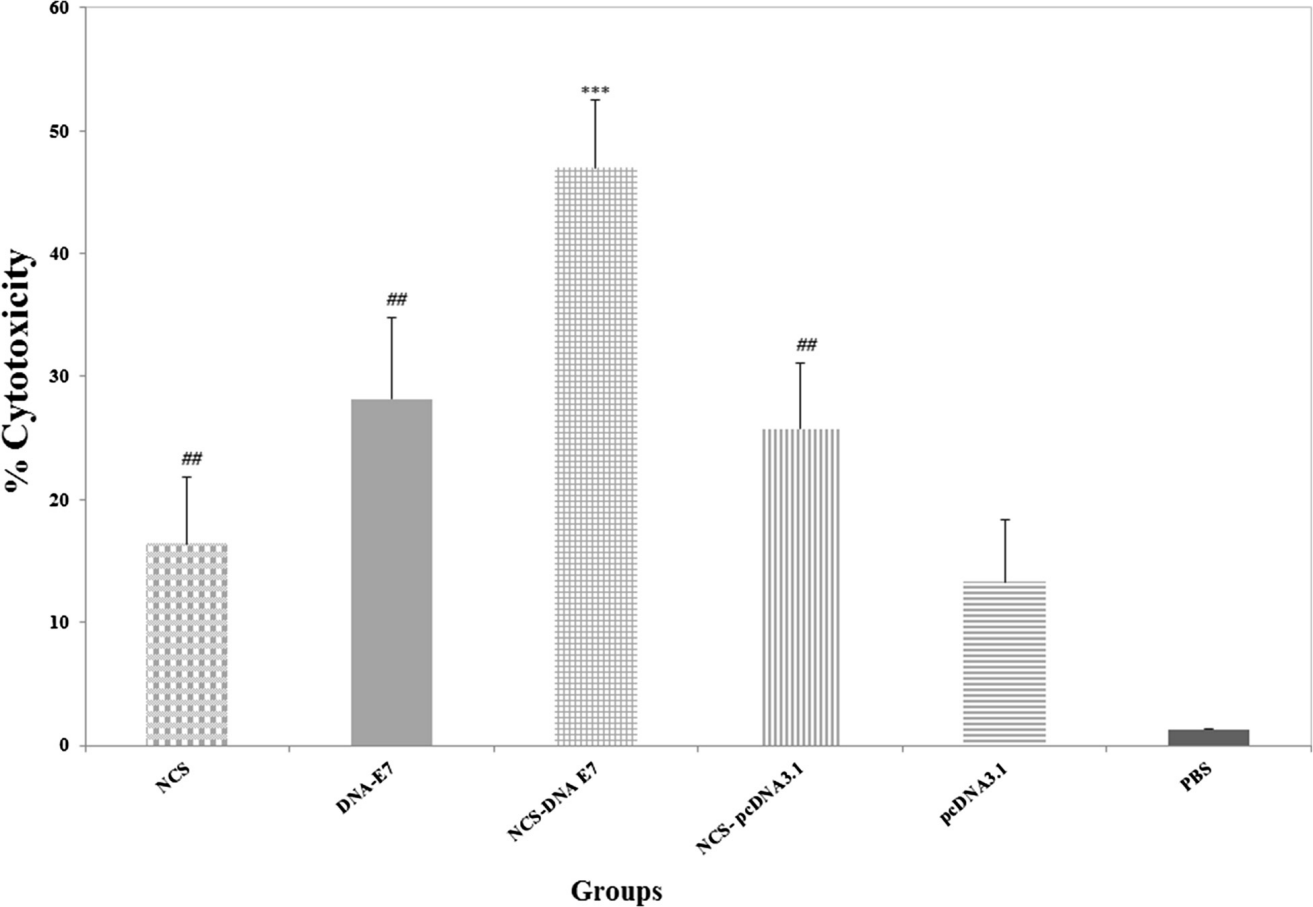
**Analysis of the cytotoxic activity of CD8 induced by NCS-DNA E7 expressing the HPV-16 E7 antigen.** CTL activity of the lymphocytes from immunized mice (five mice per group) was measured at 100:1 E/T ratio by LDH release assay kit (Takara) as described in Methods section. Specific lysis of target cells are shown with nonspecific background lysis subtracted. *** Indicates statistically significant difference between NCS-DNA E7 group as determined by one-way ANOVA (P < 0.001) with other groups. ## shows the statistical significant differences between DNA-E7, NCS-pcDNA3.1 and NCS treatments than the group treated with PBS (P < 0.01).

In order to analyze the capacity of HPV-16 E7 to enhance the E7-specific CD8 + cytotoxic T-lymphocyte (CTL) response, the response in immunized mice was examined using the LDH release assay. The LDH release increased with the E:T cell ratio up to the maximum ratio of 100:1 used in the present study. As shown in Figure 3, the cytolytic activity was significantly higher in mice treated with the NCS-DNA E7, with almost 50% specific lysis at 100:1 E/T ratio, than in those in other groups (DNA-E7, NCS, NCS-pcDNA3.1, PBS and pcDNA3.1) (P < 0.001). Furthermore, DNA-E7 (∼30% at 100:1 E/T ratio), NCS-pcDNA3.1 (∼20% at 100:1 E/T ratio) and NCS (∼17% at 100:1 E/T ratio) groups had a significantly higher antigen-specific CTL response than the group treated with PBS (P < 0.05).

### Cytokine assay

We evaluated the E7-specific IFN-γ (representative of a Th1 immune response) and IL-4 (representative of a Th2 immune response) in splenocyte culture supernatants from mice vaccinated three times with DNA vaccine encoding HPV-16 E7 with or without chitosan nanodelivery.

**Figure 4:**
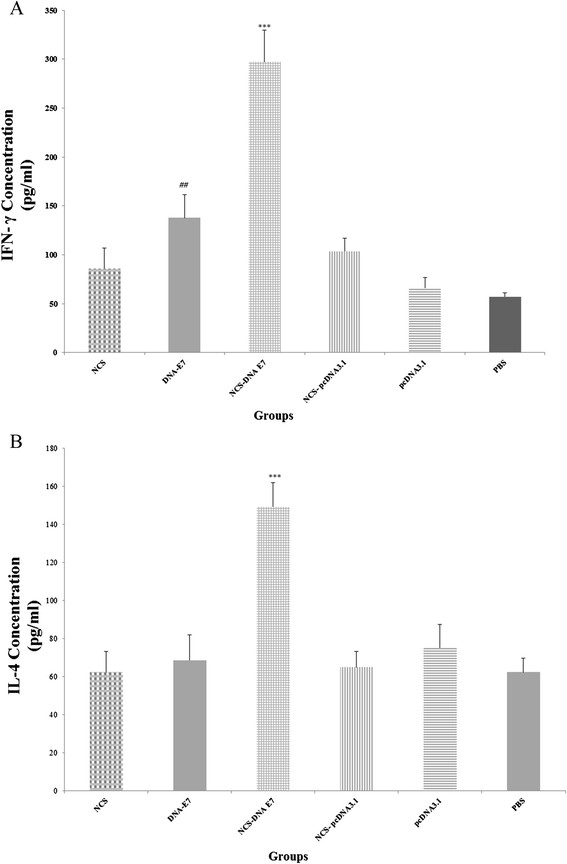
**Concentration of IFN-γ and IL-4 in supernatant following stimulation of cultured splenocytes with 4 × 10**^**5**^**TC-1 cells previously treated with mitomycin C.** Data presented as means ± standard error for five mice per group. *** Indicates statistically significant difference (P < 0.001) between NCS-DNA E7 group as determined by one-way ANOVA (P < 0.01) with other groups **(A, B)**. ## indicates the statistical significant differences between DNA-E7 treatment and pcDNA3.1 and PBS groups (P < 0.01) **(A)**.

As shown in Figure 4A, B, mice immunized with the NCS-DNA E7 produced significantly more IFN- γ and IL-4 than other immunized mice (P < 0.001) and lymphocytes from the NCS-DNA E7 group produced the largest amounts of IFN- γ and IL-4. Furthermore, the DNA-E7 vaccine stimulated IFN- γ production more than pcDNA3.1 and PBS groups (P < 0.05).

### Reduction of tumor volume by therapeutic immunization

To determine whether the observed increase in E7-specific immunity could be converted into a better E7-specific therapeutic effect, we designed an in vivo tumor therapy experiment via an E7-expressing murine tumor cell line, TC-1, as a model of cervical carcinoma.

We investigated whether administration of NCS-DNA E7 vaccine could lead to regression of preexisting tumors. For this porpuse, TC-1 cells were first injected into C57BL/6 mice at a dose of 2×10^5^TC-1 cells in the right flank. One week later, each mouse was immunized with either NCS-DNA E7, E7 DNA, NCS, NCS-pcDNA3.1, PBS and pcDNA3.1 thrice at 7-day intervals. The tumors were measured twice a week once they became palpable. The tumor volume was monitored up to 6 weeks after the tumor challenge.

**Figure 5:**
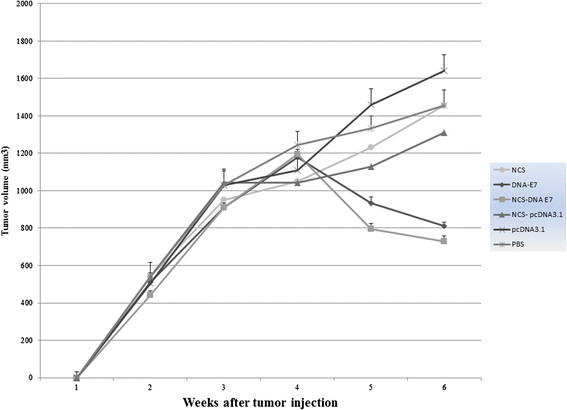
**In vivo antitumor effects generated by treatment with NCS-DNA E7, DNA-E7, NCS-pcDNA3.1 and NCS.** C57BL/6 mice were inoculated with 2×10^5^ TC-1 tumor cells subcutaneously. Mice were then treated with Nano-chitosan (NCS) and DNA regimes as described in Methods. Mice were monitored for tumor growth by measuring diameters with calipers twice a week. Line and scatter plot graphs depicting the tumor volume (in mm^3^) are presented. The data presented are a representation of three independent experiments.

As shown in Figure 5, six weeks after tumor induction, the final tumor volume were significantly reduced 52% by NCS-DNA E7 (from 1.2 to 0.8 mm^3^) and 60% by E7 DNA (from 1.2 to 0.95 mm^3^) compared to pcDNA3 or PBS control values (P < 0.001). Additionally, the average tumor volume in the NCS-DNA E7 group was significantly lower than that in the E7 DNA group (P < 0.01).

As control for nanodelivery, we also have evaluated antitumor effects of NCS and NCS-pcDNA3.1. The statistical analysis of tumor volumes did not reveal significant difference between these groups and PBS and pcDNA3.1 groups (P > 0.05).

In summary, these results showed that chitosan-based nanodelivery systems for E7 DNA vaccine could significantly reduce tumor volume and eradicate the established E7-expressing tumors. This indicated that chitosan-based nanodelivery significantly promoted antitumor immunity of DNA vaccine.

## Discussion

Cancer immunotherapy using DNA vaccines have emerged as a potentially promising approach for the control of tumors. The main purpose of this study was to develop an HPV-16 DNA vaccine that could express the E7 antigen and enhance cellular immune response compared to the naked DNA vaccine. With regard to the application of DNA vaccines, if the delivery system can be directed to APC and induce DNA vaccine release in endolysosomes, an effective T-cell immunity may be achieved at lower doses of plasmid DNA, which is safer and more cost-effective [25].

Several studies have investigated the use of chitosan as a gene delivery system, as it has shown rapid degradation in lysosomal compartment after cellular uptake, allowing for enhanced MHC-I restricted antigen presentation [4]. In mice, DNA vaccines have been delivered orally using a variety of carriers. One recent study has shown that oral gene delivery with chitosan-DNA nanoparticles was able to generate a higher level expression of gene in vivo [26].

Khatri et al. evaluated intranasal administration of chitosan pDNA nanoparticles expressing the surface antigen of hepatitis B virus (HBsAg) for immunization against hepatitis B in Balb/c mice model demonstrating successful generation of a systemic humoral and cellular immune response [27].

In addition to the oral and intranasal vaccination routes, chitosan-based systematic vaccine administration routes such as subcutaneous and intramuscular have also been explored for immunization. However, there are very limited examples where chitosan has been applied as DNA vaccine carriers for systemic vaccination.

Zaharoff et al. showed that chitosan dissolved in buffer pH 6.2 could be used as an adjuvant to enhance both cell-mediated and humoral immune responses [20]. Illum et al. evaluated a flu vaccine, formulated as a chitosan–DNA nanoparticle complex. Intramuscular or intranasal administration of the complex in BALB/c mice resulted in antibody titers that were elevated when compared to those of naked DNA [16].

In present study, we have produced NCS of fewer than 100 nm diameter to facilitate the entrance of the particles to the cells. The resultant NCS showed mean diameter of about 70 nm with spherical shape and a narrow size distribution. To evaluate the therapeutic efficacy of chitosan-based nanodelivery systems in vivo, we investigate the efficiency of intramuscular administration of encapsulated DNA vaccine in NCS, as carrier molecules for HPV-16 DNA vaccine construct, carrying the most immunogenic oncogenes of HPV-16 in mouse model for cervical cancer (TC-1 cells). The results demonstrated the induction of effective cellular immunity and antitumor response in immunized mice and supported the feasibility of Nano-chitosan to be explored for enhancing the immune responses of the DNA vaccine.

In agreement with the results of the present study, it has been previously shown that after intramuscular administration of pVAX hepatitis B virus core (HBc) DNA-nanoparticle complex as vaccine delivery systems in C57BL/6 mice, a much stronger immune response was elicited than for the vaccine alone, shown by elevated antibody production, higher level of IFN-γ secretion, and augmented Hbc Ag-specific cytotoxic T-lymphocyte response in murine splenocytes [28].

Additionally, Zhao and colleagues have showed that chitosan nanoparticles containing the DNA vaccine induced significantly swine influenza -specific cell-mediated and humoral immunity in mice [29].

Moreover, present findings are in parallel with previous studies from Zhou et al. that investigated the potency of the Chitosan as vectors for the delivery of plasmid DNA. After delivery by intramuscular immunization in BALB/c mice, the Chitosan induced an enhanced serum antibody response 10 times more potent than naked DNA vaccine. Additionally, in contrast to naked DNA, the Chitosan induced potent cytotoxic T lymphocyte responses at a low dose [30].

In other studies for evaluation of HPV-16 E7 as model antigen in the development of a therapeutic DNA vaccine candidate, several DNA vaccines against human papillomavirus (HPV) related malignancies have been performed. Our previous findings demonstrated that DNA vaccines encoding HPV-16 E7 could enhance the induction of antigen-specific cytotoxic CD8+ T cell responses and IFN-γ and confer protective immunity and therapeutic control of tumor growth [6],[12]. In a trial for anal dysplasia, encapsulated plasmid DNA encoding HLA-A2 epitopes from HPV16 E7 protein in polymer microparticles administrated intramuscularly and induced enhanced T cell responses in 10 of 12 patients [31]. Therefore, induction of both CD4+ T cells secreting Th1-type cytokines and CD8+ cytotoxic T cells play important effector roles for vaccines aiming therapeutic activation of protective antitumor immunity, similar to what was shown by the present NCS-DNA E7 vaccine. Thus, it was reasonably believed that Chitosan played a key role in supporting cellular uptake by cell–Chitosan interactions and enhanced the antigen presentation.

## Conclusions

Taken together, our study provides important insights for eradicating HPV-induced cancers exploiting a Chitosan-Based Nanodelivery. Through this new approach, the bioavailability and stability of the vaccine were significantly improved, leading to induction of antigen-specific CD8+ cytotoxic T lymphocytes (CTL), splenic CD4+. Ultimately, the delivered vaccine induced production of IFN-γ to promote antitumor immunocytotoxic response and reduce the tumor volumes.

## Abbreviations

HPV: Human papilloma virus

NCS: Nano-chitosan

CHO: Chinese hamster ovary

TPP: Pentasodium tripolyphosphate

TPP: Pentasodium tripolyphosphate

PHA: Phytohemaglotinin

APC: Antigen-presenting cell

CTL: Cytolytic T lymphocyte

IFN- γ: Interferon γ

IL-4: Interleukin 4

LDH: Lactate dehydrogenase

MTT: 3-(4,5-Dimethylthiazol-2-Yl)-2,5-diphenyltetrazolium bromide; thiazolyl-blue

OD: Optical density

FBS: Fetal bovine serum

DMSO: Dimethyl sulfoxide

RPMI: 1640 Roswell Park Memorial Institute (name of the medium)

Th: T helper

## Competing interests

All authors declare that they have no competing interests.

## Authors’ contributions

AT, did most of the experiments, AG, FA and HK participated in the design of the study, AG and AT drafted the manuscript, and AG, AT, AS and AM conceived of the study and data interpretation, MK and AS gave some suggestions. All authors read and approved the final version of the manuscript.
